# A Dedicated Lightweight Titanized Mesh Prevents Incisional Hernias After Open Abdominal Aortic Aneurysm (AAA) Repair: Results of an Initial Prospective Cohort Study

**DOI:** 10.7759/cureus.14821

**Published:** 2021-05-03

**Authors:** Akinfemi Akingboye, Arindam Chaudhuri

**Affiliations:** 1 Colorectal Surgery, Russells Hall Hospital, Dudley, GBR; 2 Vascular Surgery, Bedfordshire-Milton Keynes Vascular Centre, Bedford, GBR

**Keywords:** mesh, incisional hernia, abdominal aortic aneurysm

## Abstract

Background

Incisional hernia (IH) is a common, late complication of open repair of an abdominal aortic aneurysm (AAA), with a variable high incidence. A cohort study was conducted to investigate the role of a lightweight titanized mesh placed in the pre-peritoneal space after AAA repair. The primary endpoint was to determine the incidence of IH at eight weeks and 12 months.

Methods

Consecutive patients who underwent open repair of AAA with the prophylactic implantation of a mesh after abdominal wall closure were recruited. The development of IH was evaluated using clinical examination, ultrasonography scan (USS), and computed tomography (CT) scan during the follow-up period.

Results

Thirty-nine of 45 patients (34 male, 5 female, mean age 69.6 +/- 6.5 years) undergoing open repair of AAA over a five-year period via a preferred roof-top incision were analyzed for this study. One additional (2.5%) patient had the mesh explanted following a re-laparotomy for colonic ischemia and later developed an incisional hernia. There was no incidence of wound or mesh infection overall. One radiologically detected early IH closed spontaneously. There were five (12.8%) radiologically detected late cases of midline or paramedian defects beyond the one-year follow-up though this was not clinically significant; compared to this, there was no incidence of lateral defects in the wound (p<0.01, McNemar’s test).

Conclusion

These preliminary results suggest that a dedicated lightweight titanized mesh is usable for primary reinforcement of rooftop incisions at the time of wound closure. Whilst this study supports the role of a mesh as a useful adjunct, larger studies and long-term follow-up would provide more sensitive assessments of its efficacy.

## Introduction

Incisional hernia (IH) is an often-underestimated complication following open abdominal aortic aneurysm (AAA repair) with rates as high as 35% or more [1-3]. The evidence in recent literature points to IH occurring signiﬁcantly more often after AAA repair as compared with any other pathology, possibly owing to an underlying connective tissue disorder [4-5]. A meta-analysis has shown a ﬁve-fold increased risk of IH development in patients after open AAA repair compared with those undergoing surgery for aortic occlusive disease [4-5]. Studies have shown that the risk of IH can be reduced [2] with meticulous surgical wound closure techniques following laparotomies for major abdominal operations [5]. However, it does not completely hold true for patients undergoing repair of AAA, perhaps due to their underlying inherent genetic disorder [6].

Unfortunately, high recurrence rates have also been reported from suture repair only for incisional hernias [1-9]; hence, the use of a prophylactic primary mesh for abdominal wall closure following the open repair of AAA remains promising in the reduction of postoperative IH [2,4,10-12]. However, there appears to be insufficient evidence in the literature to cause a major paradigm shift with its use in high-risk patients. There appears to be a reluctance on the part of surgeons to change from using a midline incision to other types of non-midline incision as recommended by the European Hernia Society (EHS). This is a major rate-limiting factor in experiencing the paradigm shift. In our cohort study, we report our local experience with the use of prophylactic implantation of titanium mesh incorporated at the time of closure of rooftop incisions used for open AAA repair.

## Materials and methods

Consecutive patients undergoing elective and urgent transperitoneal AAA repair via a rooftop incision under general anesthesia with supplementary epidural analgesia were analyzed in this study. A synthetic Dacron graft (either bifurcated or tube graft) was used as appropriate and the old aortic sac was repaired around the graft. All patients received intraoperative intravenous teicoplanin 400 mg as part of the standard antibiotic regimen; they also had heparin 5000 units IV prior to the time of cross-clamping of the aorta except during repair of the ruptured AAA. The bi-layer abdominal wall closure technique was performed with polydioxanone suture using the continuous small bite technique.

**Figure 1:**
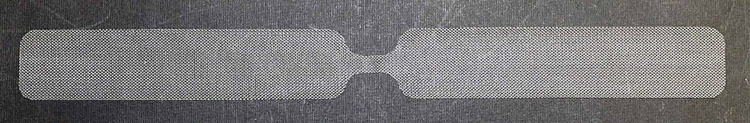
A lightweight titanized mesh (Tilene), with a central fulcrum and curved edges The central fulcrum is the only part that is stitched to the linea alba centrally and the rest of the mesh is laid in the retro-retro muscular space evenly.

**Figure 2:**
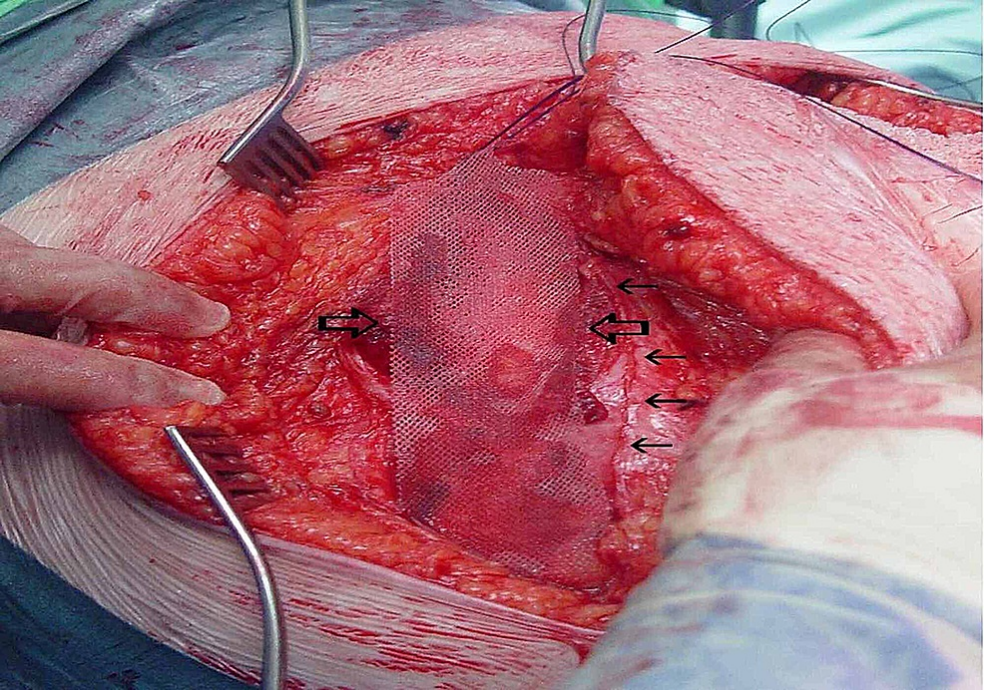
Placement of a Tilene mesh in the preperitoneal space (retro-retro rectus space) through a rooftop incision The big arrows illustrate the rectus muscle and the small arrow shows the anterior rectus sheath.

The mesh used in this study is a bridged titanized polypropylene (tPP) lightweight mesh typically used (35 g/m^2^, pore size 1000 μm) and 4x40 cm in length (Tilene® Strip, pfm Medical AG, Köln, Germany). It is characterized by minimal shrinkage and reduced inflammatory reaction as compared with the standard polypropylene mesh, which is lightweight and configured with established biocompatibility [7-8]. The mesh has a bridged portion (3x1 cm), which provides a fulcrum that allowed for bending the midline (Figure 1). A pack of three meshes costs £298 (€341.50). The mesh was placed in the sublay position, sandwiched between the posterior fascioperitoneal and anterior fasciomuscular layers, and secured centrally to the linea alba (Figure 2). Necessary precautions were taken to ensure that the mesh did not wrinkle over whilst the other layers were been closed over it. There was no need to secure the mesh at other points (thus not adding further suturing time), and the skin was closed with staples.

Clinical wound assessment was undertaken at eight weeks postoperative by the consultant vascular surgeon for all 39 patients. The incisional hernia was defined as a defect in the abdominal wall fascia either clinically or radiologically identified. The clinical examination was performed with the patient supine whilst raising both legs and/or coughing or straining in an upright position. An abdominal ultrasound scan (USS) was performed at random intervals postoperatively for the purpose of early assessment in the first round at 21, 22 weeks SD. All USS scans were undertaken by a consultant radiologist, with a special musculoskeletal interest, who was not aware of the operative background. However, after scanning the first 27 patients, discordance between the clinical evaluation and USS was perceived in (n=3 patients), with further concerns expressed by patients about such scans (n=4), hence the use of radiological assessment was abandoned. Furthermore, any other CT scan performed at any point during the second round (identified by trawling the patient radiological database) was analyzed for the purpose of identifying further development of IH; additionally, preoperative CT scans were reviewed in both the axial and sagittal view to avoid the bias created by pre-existing midline hernias. Patients not assessed radiologically had a telephonic follow-up with one of the corresponding authors and asked to self-assess for any bulges in their incision that would be indicative of an incisional hernia. Numerical and statistical analyses were undertaken using Minitab 19 software (State College, Pennsylvania).

## Results

Forty-five patients underwent elective/urgent open surgical repair (OSR) for AAA from March 2011 to December 2016. Two patients had ischaemic bowel: one patient underwent emergency re-laparotomy via the same incision for ischaemic colitis and the mesh was explanted, but later developed IH; the second had a midline laparotomy and thus did not have the mesh explanted. Six patients were eventually unavailable for analysis, including the one patient with mesh explant, one refusal to undergo radiological follow-up, and four 30-day mortalities. Thirty-nine patients (34 male, 5 female, age 68.8+/-6.8 years; body mass index (BMI) 27.1+/-4.2) were therefore available for analysis as presented below (Table 1), though all survivors were reviewed in the outpatient clinic at six to eight weeks postoperatively. The AAA size was a mean 65.6+/-13.4 mm. Three (6.8%) patients had isolated iliac aneurysms repaired via the rooftop incision. One patient was commenced as endovascular aneurysm repair (EVAR) but successfully converted to open repair because of device failure. There was no incidence of mesh infection or any superficial or deep wound infection over both rounds. One patient had superficial wound dehiscence, successfully managed with secondary suturing. The postoperative length of hospital stay was mean 9+/-3 days. There were two (5.1%) cases of pre-existing umbilical hernia and two (5.1%) cases of inguinal hernia (both bilateral) reported as incidental findings on USS and correlated on CT. Of the first 31 consecutive patients operated on, four did not have their scan because of concerns that they may require further treatment; clinically they did not have any incisional hernias. Of the remaining 27, 24 had USS done and three had a CT scan. In the first round, only one patient (2.6%) had a midline/paramedian defect, which had healed by the next scan at 34 weeks.

**Table 1:** Summarizing the patient’s demographic data, the morphology of the abdominal aortic aneurysm, and the type of aortic surgery and patient’s risk factors BMI: body mass index; AAA: abdominal aortic aneurysm

Parameters	Value
Gender	Male 34/39 (87.2%); Female 5/39 (12.8%)
Age (mean ± SD)	69.6± 6.5
BMI (mean± SD)	27.1 ± 4.2
Diabetic patients	3/39 (7.7%)
Type of Aneurysm: Infra-renal; Juxta-renal; Iliac aneurysm	35/39 (89.7%); 1/39 (2.7%); 3 /39 (7.6%)
AAA Morphology: Inflammatory; Non-inflammatory; Operation group: Rupture repair; Urgent repair; Elective repair	5/39 (12.8%); 34/39 (87.2%); 3/39 (7.7%); 9/39 (23.1%); 27/39 (69.2%)
Length of Stay (days; mean ± SD)	9±3
Type of Graft: Tube; Bifurcated	31/39 (79.5%); 8/39 (20.5%)
Incidence of incisional hernia; At 8 weeks	No incisional hernia; Incidental hernia detected on USS 5/39 (12.8%); umbilical hernia 2/39 (5%); inguinal hernia
Mesh Explantation	1/39 (2.6%) (following laparotomy for ischaemic bowel)
Average Follow-Up	12 months
Return to Theater	1/39 (2.7%); Laparotomy for ischaemic bowel and mesh explantation

Only 15 of these 27 patients had any form of secondary radiology follow-up in the second round (USS, n=12, CT, n=3; time to scan at 103+/-58 weeks), where a further five (12.8%) patients had small median or paramedian defects noted; three of these had no content protrusion, one had an omental protrusion, and one had extraperitoneal fat protrusion; no patient needed these repaired. The occurrence of these defects did not correlate with BMI (Pearson correlation 0.041, p>0.8). However, there were no defects noted laterally clinically in all patients, and this was significantly lower as compared to the number of midline defects (p< 0.001, McNemar’s test). The remaining patients did not report any obvious bulges on telephonic follow-up.

## Discussion

The incidence of IH after midline laparotomy post-AAA repair is a common, long-term complication (>35%). This incidence seems high when compared with the incidence of IH post-laparotomy for other major intra-abdominal operations [13-17]. A systematic review by Takagi et al. reported that incisional hernia is more common with AAAs as compared to aortic occlusive disease with an incidence of 21% and 9.8% over a relatively similar length of time [4]. Louridas et al reported an increased incidence of IH of 29.1% at 24 months of follow-up and a five-year incidence of 69.1% for open repair of AAA [18]. It has been suggested that the systemic proteolytic effect and the increased turnover of type III collagen might play an important role in the development of IH in patients having AAA repair [19]. There appears to be a commonality between AAA and abdominal wall hernia, which is characterized by a dysregulation of the proteolytic activity and the disruption of the protease system [20-21]. This, in turn, results in an abnormal connective tissue remodeling process and disorganization of collagen synthesis [10]. Contrary to the traditionally known risks (obesity, diabetes, chronic lung disease, and smoking), evidence from molecular studies has shown that these factors are of less relevance in the etiology of IH in open aortic aneurysm repair [21]. The published figures may have underestimated the true incidence, perhaps because of short-term follow-up or because most symptomatic patients with IH present to another clinician.

In our series, the mean follow-up period was 12 months, which we accept may not be sufficiently long enough to see an incisional hernia develop, which is a limitation of this study. However, Deerenberg et al., in a prospective, multicentre, double-blind, randomized controlled trial over a years' period were able to demonstrate the incidence of incisional hernia following the closure of the laparotomy wound using either the large or small bites closure technique [22]. Most of the studies reporting incidences of incisional hernia post-aortic reconstructive surgery had less than a five-year follow-up. The majority of significant incisional hernias develop in the first two years following surgery, which incidence increases as the year advances, with an increase in the incidence of up to 35% occurring five to 10 years following surgery [23].

It is an established fact that the use of ancillary diagnostic imaging techniques, such as USS, CT scan, and magnetic resonance imaging, recommended by the European Hernia Society for the detection of incisional hernia post laparotomy will lead to an increase in the reported incidence of incisional hernia as compared to clinical evaluation alone [9]. However, there is no consensus about the superiority of any of the imaging modalities over the other. In our study, given the discordance between the findings of the clinical evaluation and the USS scan results, which meant that there was a possible overdiagnosis of abdominal wall defects; the use of USS was discontinued. A CT scan has the advantage of a higher predictive value and specificity, as well as a better intra-and inter-observer reliability in diagnosing incisional hernia over USS [14,23]. Our results show that a CT scan is a means to resolve the disparity. This is one of the study limitations; our preferred choice would have been to perform CT scans on all our patients postoperatively, at six and 12 months respectively.

A recent systematic review conducted by Bickenback et al. [16] and a Cochrane review by Brown et al. [15] concluded that non-midline incisions signiﬁcantly reduced the risk of incisional hernia as compared to midline incisions, but did not inﬂuence the risk of a burst abdomen. There is no debate that the incidence of incisional hernia is higher in longitudinal midline incisions than transverse, hence, the European Hernia Society has also advocated alternate incisions to midline laparotomy, perhaps a transverse abdominal incision, during any elective major abdominal surgery [9]. In addition, a possible reduced length of hospital stay and an improved postoperative course has been linked with a short transverse incision, though there is no strong convincing evidence on this subject; hence, the recommendation from the European Society for Vascular Surgery on the management of AAA is that the incision should be tailored to the patients' needs and local expertise [24]. This recommendation provides the template for the change in our practice; as a default, we use a rooftop incision for elective AAA repair and, where feasible, for stable patients requiring emergent AAA repair, including ruptured AAA.

Our study is unique in that it employed the use of rooftop incision with the incorporation of a tPP mesh, which has not been previously described. Although there are no published studies to date detailing the incidence of incisional hernia following rooftop incision, we can only hypothesize that the incidence of incisional hernia will be lower compared to the other two popular incisions (midline vertical and transverse incision). Our study is highly supportive of other published [2,4,9,12,17] literature and gives credence to existing evidence that the incidence of incisional hernia is lower with the implantation of a prophylactic mesh during high-risk abdominal operations such as AAA repair. We reported one of the lowest incidence rates published (5%) on incisional hernia post-AAA, which is attributable to the type of the incision used and perhaps due to the type of mesh.

Furthermore, the mesh has the advantage of easy adsorption into a thin ﬁbrous tissue layer, with cells growing through the pores of the mesh fabric, which results in the complete enclosure of the mesh material by the patient’s own body tissue. In addition, the tPP mesh is a non-resorbable material, which is not affected by infections or contamination [6,19]. Another added advantage with the use of tPP is that the technique for mesh fixation does not prolong the operation or add significant time to wound closure. The mesh is fixed is the midline and the only step required was passing the sutures through the mesh, which takes a few seconds, followed by closing the anterior musculoaponeurotic layer superficial to it. This perhaps explains the very low incidence of wound infection or mesh explantation in our series. There are no published comparative studies between different mesh types, mesh position, or method of mesh ﬁxation in preventing incisional [9-10]. In the systematic review and meta-analysis conducted by Wang et al., they showed that prophylactic mesh reinforcement can effectively decrease the incidence of IH and overall improved the quality of life [24]. However, in their study, no demonstrable differences in postoperative overall morbidity, systemic postoperative morbidity, wound-related morbidity, surgical site infection, hematoma, wound disruption, postoperative mortality, and length of hospital stay in the mesh and non-mesh groups were compared [22,25]. Contrary to our experience, they reported a higher rate of seroma formation and increased operative time as a consequence of prophylactic mesh implantation.

In a single-center study published by Caro-Tarrago et al. [12], where the mesh was placed in an on-lay position, a high incidence of seroma formation was noticed. In our study, there were no cases of seroma detected; perhaps, it may be attributed to the preperitoneal mesh placement technique and less inflammatory response produced with the tPP mesh [10,19]. The use of a rooftop incision and implantation of the tPP mesh did not appear to significantly increase operating time. Although the study was not set out to estimate the time of mesh implantation because once the mesh is laid flat in the preperitoneal space (retro-muscular plane), the mesh is simply secured at the fulcrum to the linea alba at the midpoint with no other stitching required to secure the mesh.

The overall conclusion from the European Hernia Society consensus and guidelines was that there is no optimum position for mesh placement, fixation technique, or the type of prophylactic mesh used for augmentation. This was a result of a lack of sufficient published evidence. However, it appears as though prophylactic mesh augmentation is effective and safe in high-risk patients for the prevention of incisional hernias [9]. The present study demonstrates that the reinforcement of rooftop incision with a bridged titanized polypropylene mesh is safe, with no major adverse effect. Following the outcome from the pilot study, most of the vascular surgeons in the hospital now adopt this technique. This study is unique in that it contributes a novel technique to the closure of abdominal wall incision with the potential of reducing the incidence of incisional hernia.

Although this pilot study has highlighted the benefits of the prophylactic use of wound augmentation with the use of mesh to reduce the development of IH, there other flaws in the study such as the lack of a group comparison, the non-uniformity with the use of radiological assessment techniques, which meant missing some clinically unapparent hernias. However, from a practical standpoint, it may be moot, as these patients did not need any further repairs. The timing of interval assessment for the hernias should equally be standardized, which has a linear correlation with the development of postoperative hernias. The combination of clinical evaluation and self-examination by patients in no doubt introduced inconsistency in the detection of IH. Furthermore, the combination of a structured telephone questionnaire directed at self-assessment by the patient was a novel idea by the author, which aimed to prevent unnecessary hospital visits in a group of elderly patients with multiple co-morbidities. In hindsight, the current COVID pandemic has, in fact, clearly created exactly such a situation where face-to-face consultations now are the exception and not the rule. We nevertheless acknowledge that this indeed is a limitation, given the mixed-modality of the follow-up approach, which clearly introduced a bias element.

## Conclusions

Prophylactic/primary preperitoneal wound augmentation using a dedicated “bridged” lightweight tPP mesh at abdominal rooftop incision closure after OSR for AAA is safe and convenient. Whilst this study indicates such an adjunct may help reduce early IH formation after open AAA repair, larger studies, and prolonged follow-up would provide more robust assessments of its long-term efficacy. This is currently difficult given the uptake of endovascular repair in preference to OSR. The midline remains vulnerable despite mesh supplementation, though it is clinically insignificant in this series, and this may be because the bridged section of the mesh is thinner than the rest.
